# Multiple Large Language Models’ Performance on the Chinese Medical Licensing Examination: Quantitative Comparative Study

**DOI:** 10.2196/77978

**Published:** 2025-12-16

**Authors:** Yanyu Diao, Mengyuan Wu, Jingwen Xu, Yifeng Pan

**Affiliations:** 1 The School of Big Data and Artificial Intelligence, Anhui Xinhua University, 555 Wangjiang West Road, Hefei, 230088, China, 86 15905667742

**Keywords:** ChatGPT, Chinese National Medical Licensing Examination, medical student, ERNIE Bot, Tongyi Qianwen, artificial intelligence, AI

## Abstract

**Background:**

ChatGPT excels in natural language tasks, but its performance in the Chinese National Medical Licensing Examination (NMLE) and Chinese medical education remains underexplored. Meanwhile, Chinese corpus–based large language models (LLMs) such as ERNIE Bot, Tongyi Qianwen, Doubao, and DeepSeek have emerged, yet their effectiveness in the NMLE awaits systematic evaluation.

**Objective:**

This study aimed to quantitatively compare the performance of 6 LLMs (GPT-3.5, GPT-4, ERNIE Bot, Tongyi Qianwen, Doubao, and DeepSeek) in answering NMLE questions from 2018 to 2024 and analyze their feasibility as supplementary tools in Chinese medical education.

**Methods:**

We selected questions from the 4 content units of the NMLE’s General Written test (2018‐2024), preprocessed image- and table-based content into standardized text, and input the questions into each model. We evaluated the accuracy, comprehensiveness, and logical coherence of the responses, with quantitative comparison centered on scores and accuracy rates against the official answer keys (passing score: 360/600).

**Results:**

GPT-4 outperformed GPT-3.5 across all units, achieving average accuracies of 66.57% (SD 3.21%; unit 1), 69.05% (SD 2.87%; unit 2), 71.71% (SD 2.53%; unit 3), and 80.67% (SD 2.19%; unit 4), with consistent scores above the passing threshold. Among the Chinese models, DeepSeek demonstrated the highest overall performance, with an average score of 454.8 (SD 17.3) and average accuracies of 73.2% (unit 1, SD 2.89%) and 71.5% (unit 3, SD 2.64%), as well as average accuracies of 70.3% (unit 2, SD 3.02%) and 78.2% (unit 4, SD 2.47%). ERNIE Bot (mean score 442.3, SD 19.6; unit 1 accuracy =70.8%, SD 3.01%; unit 2 accuracy =68.7%, SD 3.15%; unit 3 accuracy =69.1%, SD 2.93%; unit 4 accuracy =68.3%, SD 2.76%), Tongyi Qianwen (mean score 426.5, SD 21.4; unit 1 accuracy =67.4%, SD 3.22%; unit 2 accuracy =65.9%, SD 3.31%; unit 3 accuracy =66.2%, SD 3.08%; unit 4 accuracy =67.2%, SD 2.89%), and Doubao (mean score 413.7, SD 23.1; unit 1 accuracy =65.2%, SD 3.45%; unit 2 accuracy =63.8%, SD 3.52%; unit 3 accuracy =64.1%, SD 3.27%; unit 4 accuracy =62.8%, SD 3.11%) all exceeded the passing score. DeepSeek’s overall average accuracy (75.8%, SD 2.73%) was significantly higher than those of the other Chinese models (χ²₁=11.4, P=.001 vs ERNIE Bot; χ²₁=28.7, P<.001 vs Tongyi Qianwen; χ²₁=45.3, P<.001 vs Doubao). GPT-4's overall average accuracy (77.0%, SD 2.58%) was slightly higher than that of DeepSeek but not statistically significant (χ²₁=2.2, P=.14), while both outperformed GPT-3.5 (overall accuracy =68.5%, SD 3.67%; χ²₁=89.8, P<.001 for GPT-4 vs GPT-3.5; χ²₁=76.3, P<.001 for DeepSeek vs GPT-3.5).

**Conclusions:**

GPT-4 and Chinese-developed LLMs such as DeepSeek show potential as supplementary tools in Chinese medical education given their solid performance on the NMLE. However, further optimization is required for complex reasoning, multimodal processing, and dynamic knowledge updates, with human medical expertise remaining central to clinical practice and education.

## Introduction

Natural language processing (NLP) is an artificial intelligence (AI) technology that aims to allow computers to understand, process, and generate natural language [1]. ChatGPT is the most successful commercial model of NLP technology that enables high-quality natural language comprehension and generation, and its core algorithm is a transformer, a deep neural network structure based on a self-attentive mechanism with strong sequence modeling capabilities and representation learning [2-4]. Through the process of pretraining and fine-tuning, the ChatGPT model can understand and generate natural language text and be useful in various application scenarios, such as automatic question answering, intelligent customer service, speech recognition, and machine translation. The success of the ChatGPT model is attributed to the excellent performance of the transformer and the maturity of the pretraining technique. In the pretraining phase, the ChatGPT model can learn the patterns and features of language from large-scale text data through unsupervised learning, allowing the model to be fine-tuned on a limited dataset and achieve excellent performance [5-7].

Considering that ChatGPT is an evolving NLP model that has been successfully used in many fields, several medical education research groups have been investigating its feasibility as a valuable tool for clinical support and medical education. Gilson et al [8] applied ChatGPT to the US Medical Licensing Examination, and the experimental results showed that it met the passing criteria. However, Huh [9] found in their study that, during a Korean parasitology examination, 77 out of 79 medical students performed better than ChatGPT. Specifically, ChatGPT’s scores were lower than those of the medical students, and its correct response rate showed no correlation with the knowledge difficulty level of the examination items. Although the version of ChatGPT at that time was still at GPT-3.5 and not the state-of-the-art GPT-4, questions were raised about the ability of ChatGPT to provide medically accurate answers when using non-English languages, especially Chinese [10].

Therefore, to explore the capability of large language models (LLMs) in medical education in Chinese, in addition to applying GPT-3.5 and GPT-4, developed by OpenAI, this study applied ERNIE Bot [11], developed by Baidu; Tongyi Qianwen [12], developed by Alibaba Cloud; Doubao (ByteDance); and DeepSeek to evaluate the feasibility of LLMs and compare multiple LLMs and the advantages and disadvantages of multiple bigrams in Chinese medical education. This is considering that ERNIE Bot, Tongyi Qianwen, Doubao, and DeepSeek, being deeply rooted in the Chinese language environment, use large and rich Chinese datasets in the training process, which may endow them with stronger Chinese comprehension than that of ChatGPT. However, the advantages and disadvantages of multiple LLMs for Chinese medical education need to be corroborated through experiments.

While previous studies have explored the potential of LLMs in medical education, their performance in the context of the Chinese National Medical Licensing Examination (NMLE) remains largely unexplored. As a rigorous and standardized assessment that determines the eligibility of medical professionals in China, the NMLE presents unique challenges due to its specific focus on Chinese medical knowledge, cultural context, and clinical practices [13,14]. Given that existing research on language models in medical education has primarily centered on Western-based examinations and English-language models, there is a critical need to evaluate how models such as GPT-3.5, GPT-4, ERNIE Bot, Tongyi Qianwen, Doubao, and DeepSeek perform in this distinct Chinese medical assessment environment. By systematically comparing these models’ performance on NMLE questions, this study aimed to fill a significant gap in the literature, providing empirical evidence on the feasibility of LLMs in Chinese medical education. The findings of this research will not only contribute to the understanding of how these models can be integrated into Chinese medical curricula but also offer valuable insights for enhancing the education and training of future medical professionals in China. The results may offer preliminary insights into the potential applications of these models in Chinese medical education and contribute to ongoing discussions on integrating such technologies into medical curricula.

## Methods

The dataset used in this study comprised the original Chinese NMLE test questions from 2018 to 2024. Each annual examination consisted of 4 units, as delineated in Table 1, with each unit containing 150 multiple-choice questions, adding up to a total score of 600 points. A minimum score of 360 is required to pass the examination. Given the varying capabilities of LLMs in processing multimodal content, a protocol was established, which is outlined in this section.

**Table 1.** Exam content and scores for the 4 units of the Chinese National Medical Licensing Examination.

Unit	Maximum possible score	Exam content
1	150	Infectious diseases, psychoneurology, endocrinology, pharmacology, physiology, biochemistry, medical regulations, medical ethics, medical microbiology, medical psychology, preventive medicine, and medical immunology
2	150	Infectious diseases, pathology, psychiatry, endocrinology, respiratory medicine, urology, digestive medicine, cardiovascular medicine, hematology, physiology, biochemistry, exercise science, and medical immunology
3	150	Psychiatry, urology, gastroenterology, cardiovascular medicine, hematology, symptoms and signs, sports medicine, and pharmacology
4	150	Female reproductive system, pediatrics, and psychiatry

This study evaluated six LLMs with distinct capabilities for handling visual content.

1. GPT-3.5: lacks native image processing capabilities.
2. GPT-4: supports limited image input in specific use scenarios (eg, direct image insertion in its official interface), but for the standardized testing in this study—where all models were evaluated under uniform text input conditions to eliminate variability from different multimodal processing capabilities—visual elements were manually converted to descriptive text.
3. ERNIE Bot: includes optical character recognition functionality but was tested using text-based descriptions to ensure consistency.
4. Tongyi Qianwen: a multimodal version exists, but the internal test version used relied on text inputs.
5. Doubao: it is primarily text based; visual content was converted to descriptive text.
6. DeepSeek: while it is advanced in medical reasoning, visual elements were preprocessed into text to maintain uniform evaluation criteria.

To ensure equitable comparison, all image- and table-based questions across units were preprocessed using the following methods:

1. Structured tables were converted into CSV format with headers (eg, “Parameter,” “Value,” and “Unit”).
2. For medical images, all descriptions (including chest x-rays, computed tomography screenshots, and pathological section diagrams) in this study were generated based on the 3 elements of clinical standard terminology+visual key features+diagnostic correlation information. Taking chest x-ray images as an example, a description template was used (“[Examination type] shows the presence of [visual features, such as patchy shadows/nodules/fluid accumulation] in [anatomical location], accompanied by [associated clinical information, such as blurred boundaries/mediastinal displacement], consistent with [common disease indications, such as typical manifestations of community-acquired pneumonia]. Before generation, extract the official reference answers and analysis of the image questions in the NMLE real test, ensuring that the description does not contain direct diagnostic conclusions (only objectively presenting visual information), and avoiding premature disclosure of answers. The image description was independently completed by 2 attending physicians with more than 5 years of clinical experience (both holding Chinese physician qualification certificates and specializing in respiratory medicine and radiology, respectively), and then cross-reviewed by a deputy chief physician (affiliated with the clinical imaging diagnosis department). If there were differences in the descriptions of the 2 attending physicians (such as inconsistent expressions of “shadow density”), a consensus was reached through 3-person negotiation to ultimately form the “NMLE Image Question Text Description Manual,” ensuring the objectivity, consistency, and clinical accuracy of all descriptions.
3. Diagrams were translated into step-by-step textual explanations (eg, “Flowchart depicting the diagnostic pathway for diabetes mellitus”).

**Figure 1.**
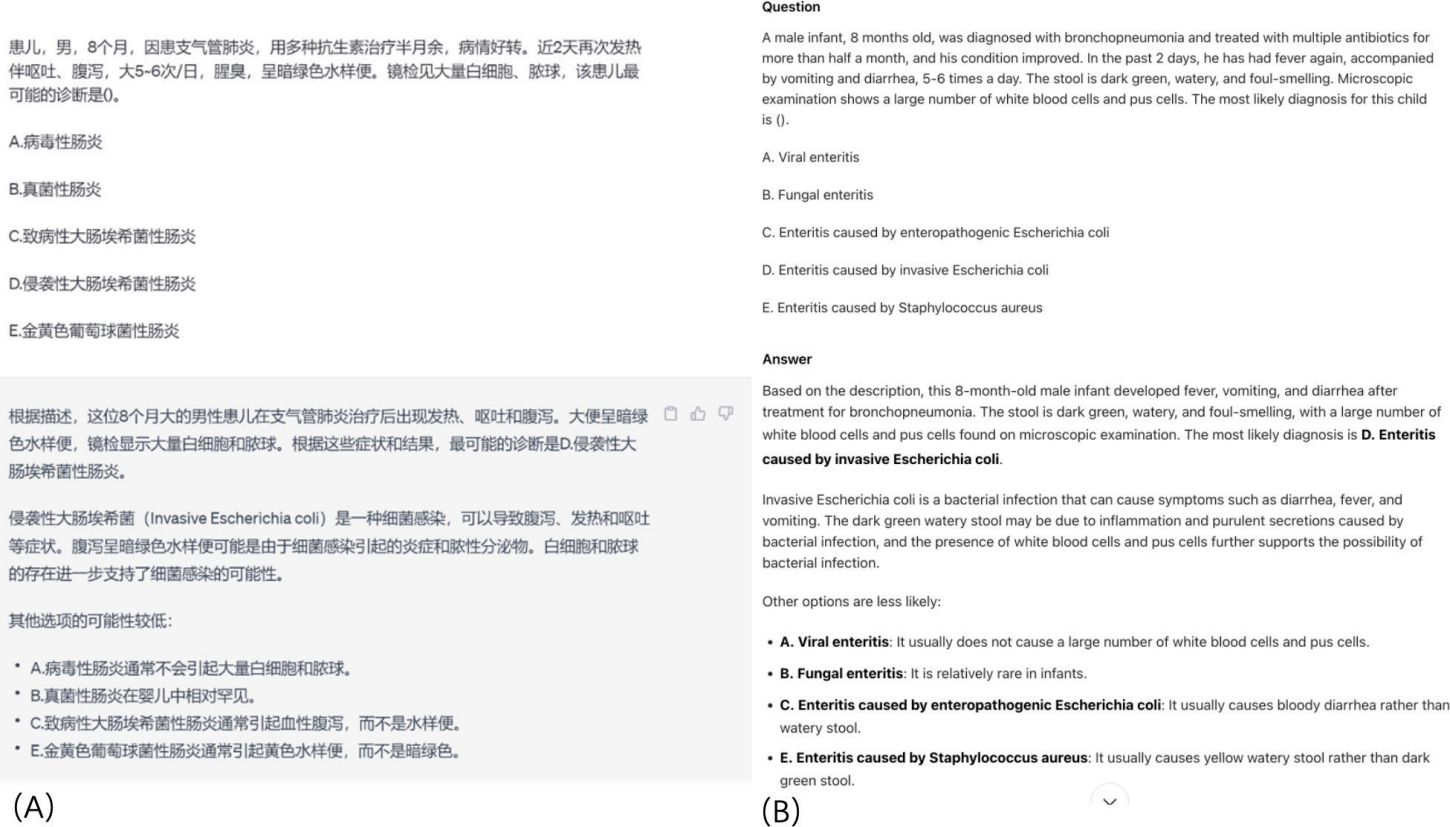
Test question input process: (A: Chinese, B: English translation).

Each model was tested using the same protocol: preprocessed NMLE questions from 2018 to 2024 were systematically input, and responses were recorded. To ensure consistency in the experimental conditions, each NMLE question (after preprocessing) was prompted once to each model—no repeated prompting was conducted for the same question as repeated inputs might lead to inconsistent response adjustments by the models, which would interfere with the accuracy of the performance comparison. Regarding the parameter settings of the models: for GPT-3.5 and GPT-4, the temperature parameter was set to 0.2, and the top-p parameter (also known as nucleus sampling, a parameter that controls the diversity of model outputs by limiting the selection range to the smallest set of tokens whose cumulative probability exceeds the specified value) was set to 0.95; these settings were chosen to balance the stability and accuracy of the models’ responses—temperature of 0.2 reduces the randomness of the generated content to avoid arbitrary answers, whereas top-p of 0.95 ensures that the models still retain a moderate range of token selection to cover potential correct reasoning paths. For the Chinese models (ERNIE Bot, Tongyi Qianwen, Doubao, and DeepSeek), we used their default parameter configurations (consistent with the settings in their official public interfaces for general question answering tasks) as their internal adjustable parameter details (eg, specific temperature and top-p ranges) were not publicly disclosed by the developers at the time of the study. All parameter settings and prompting rules were recorded in the study’s experimental log to ensure the reproducibility of the results. Answers were scored against the official answer keys. The input process, standardized for all models, is illustrated in Figure 1.

## Results

### Comparison of GPT-3.5 and GPT-4

This study analyzed the scores of GPT-3.5 and GPT-4 in the NMLE exams from 2018 to 2024. The results are outlined in this section.

**Figure 2.**
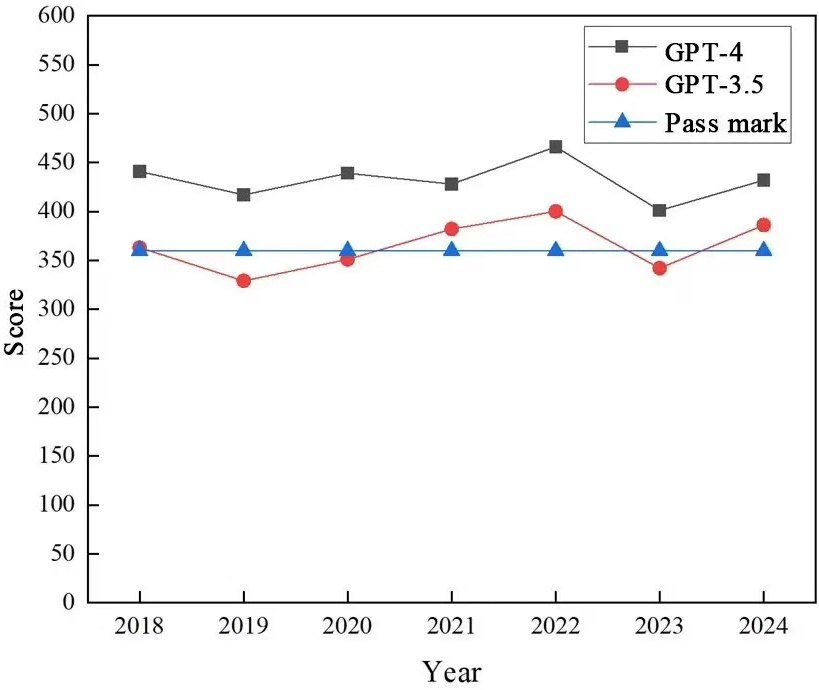
GPT-3.5 and GPT-4 scores in the 2018 to 2024 Chinese National Medical Licensing Examination.

As shown in Figure 2, GPT-4, represented by the gray squares, consistently surpassed the pass mark (set at 360 points, indicated by the blue-triangle series) across the 7 examination years. From 2018 to 2024, scores exhibited fluctuations (2018: 441; 2019: 417; 2020: 439; 2022: 466 [peak]; 2023: 401; 2024: 432). GPT-3.5, indicated by the red circles, had a more inconsistent performance. While it passed the exam in 2018 (scoring 363), 2020 (351), 2021 (382), 2022 (400), and 2024 (386), it fell below the pass mark in 2019 (329) and 2023 (342).

Overall, GPT-4 demonstrated a more stable ability to meet or exceed the passing criteria in the NMLE exams over the 7-year period compared to GPT-3.5.

Considering that the test questions of the NMLE exam consist of 4 units, the content of each unit of the exam is highly differentiated. Therefore, this study used GPT-3.5 and GPT-4 for different units and counted their score rates.

Table 2 compares the score performance and average accuracy of GPT-3.5 and GPT-4 across different units of the Chinese NMLE from 2018 to 2024. The data reveal that GPT-4 outperformed GPT-3.5 comprehensively in all units and years. Specifically, for units 1 to 4, GPT-4 achieved average accuracies of 66.57%, 69.05%, 71.71%, and 80.67%, respectively, which were higher than GPT-3.5’s accuracies of 61.24%, 58.67%, 56.86%, and 66.38%, respectively. To verify the statistical significance of this performance difference, we conducted a chi-square test on the correct and incorrect answers of GPT-4 and GPT-3.5 across all 4200 questions (2018‐2024 NMLE) with a significance threshold of *P*<.05. The results showed that GPT-4’s accuracy was significantly higher than that of GPT-3.5 in all units (unit 1: χ²₁=12.4, df=1; P<.001, unit 2: χ²₁=15.7, df=1; P<.001, unit 3: χ²₁=19.0, df=1; P<.001, and unit 4: χ²₁=23.1, df=1; P<.001). This indicates that GPT-4 exhibits stronger capabilities in understanding and applying medical knowledge, and the performance gap between the 2 models was statistically significant.

**Table 2.** Comparison of GPT-3.5 and GPT-4 scores in different units.

	GPT-3.5				GPT-4			
	Unit 1	Unit 2	Unit 3	Unit 4	Unit 1	Unit 2	Unit 3	Unit 4
2018 score	88	92	81	102	102	99	112	128
2019 score	78	84	74	93	97	102	100	118
2020 score	83	80	76	112	89	107	113	130
2021 score	102	91	86	103	106	98	104	120
2022 score	98	97	101	104	101	113	118	134
2023 score	92	83	80	87	94	92	111	104
2024 score	102	89	99	96	110	114	95	113
Accuracy (%)	61.24	58.67	56.86	66.38	66.57	69.05	71.71	80.67

Regarding the annual score patterns, neither GPT-3.5 nor GPT-4 showed a predictable year-on-year increase in performance, which aligns with the expectation that model performance is not inherently tied to the exam year. Instead, the score fluctuations observed for both models (eg, GPT-3.5’s 14-point and 6-point declines in unit 2 and unit 3, respectively, in 2023 compared to the previous year and GPT-4’s 24-point drop in unit 4 in 2023) primarily reflect variations in exam difficulty across years rather than inherent changes in model capability. Importantly, neither model’s performance showed a substantial decline across the 2018 to 2024 period relative to their initial performance, indicating that the models likely did not encounter or memorize the specific NMLE questions during their training (given the training cutoff periods before the later exam years). While GPT-4 did not exhibit a clear upward trend, its fluctuations were more moderate than those of GPT-3.5, and it maintained consistent performance above GPT-3.5 across all years—with unit 4 showing a 5-point difference between its 2018 and 2024 scores—further reflecting its stronger stability in adapting to the varying content and difficulty of complex medical scenarios.

Moreover, the 2 models demonstrated distinct performances across different units, with statistical significance verified via chi-square tests (P<.05) on their correct and incorrect answers across all questions in each unit. GPT-3.5 showed relatively better performance in unit 4, although the difference was less pronounced (χ²₁=4.2, df=1; P=.04), whereas GPT-4 had a clear and statistically significant edge in both unit 3 (χ²₁=27.6, df=1; P<.001) and unit 4 (χ²₁=19.9, df=1; P<.001). This variation may be related to the differences in the content focus of each unit: unit 3 emphasizes clinical reasoning and comprehensive case analysis, which aligns with GPT-4’s strengths in complex logical deduction, whereas unit 4 covers basic medical knowledge, which is more accessible to both models.

To contextualize the performance of the tested LLMs, it is necessary to reference the human score data of the NMLE from 2018 to 2024 as publicly reported by China’s National Medical Examination Center. During this period, the average score of human examinees (primarily medical graduates and practicing physicians) in the NMLE General Written test ranged from 420 to 455 points (out of 600), with an average accuracy rate of 70% to 75.8%. Specifically, in 2018, the average human score was 425 points (70.8% accuracy); in 2020, it was 432 points (72% accuracy); in 2022, it was 448 points (74.7% accuracy); and in 2024, it was 452 points (75.3% accuracy). GPT-4 (mean score 435.3 points; 72.6% accuracy) showed comparable overall performance to that of average human examinees, whereas DeepSeek (mean score 454.8 points; 75.8% accuracy) slightly exceeded the average human score. In contrast, GPT-3.5 (mean score 373.3 points; 62.2% accuracy) and Doubao (mean score 413.7 points; 68.9% accuracy) remained below the average human performance. Notably, top-performing human examinees (top 10% of test takers) consistently achieved scores above 510 points (85% accuracy), a threshold that no tested LLM reached in this study.

### Comparison of ERNIE Bot, Doubao, DeepSeek, and Tongyi Qianwen in the NMLE (2018-2024)

**Figure 3.**
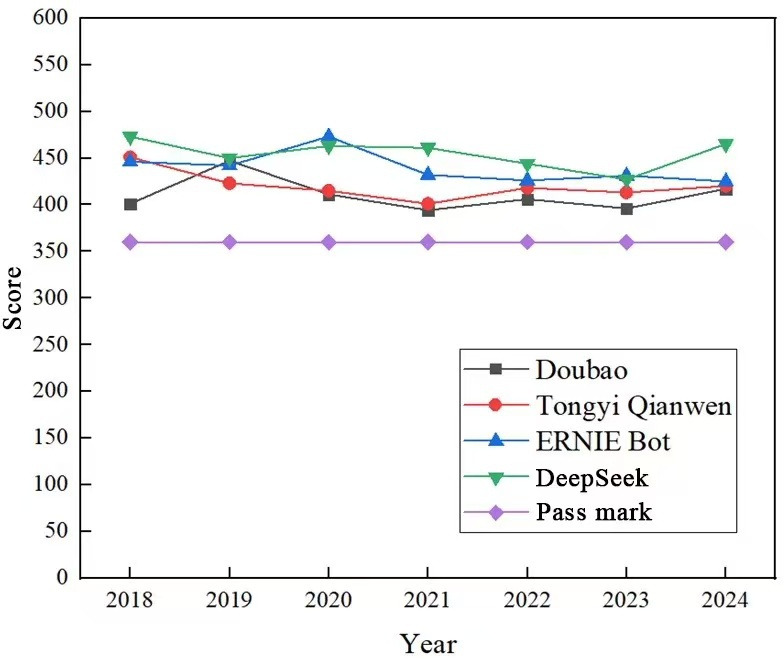
Comparison of ERNIE Bot, Doubao, DeepSeek, and Tongyi Qianwen in the Chinese National Medical Licensing Examination (2018-2024).

The comparative analysis of these 4 LLMs revealed distinct performance patterns across the 7-year period (2018-2024). As shown in Figure 3, DeepSeek consistently outperformed its counterparts in most examination years, demonstrating remarkable stability in medical knowledge assessment.

Among the 4 Chinese LLMs, DeepSeek achieved the highest overall average score across the NMLE in 2018 to 2024, with a mean score of 454.8, which was notably higher than those of ERNIE Bot (442.3), Tongyi Qianwen (426.5), and Doubao (413.7). Further analysis of performance across exam units (reflecting different medical topic domains) revealed that DeepSeek exhibited consistent strengths in unit 1 (medical ethics, regulations, and basic medical sciences) and unit 3 (comprehensive clinical reasoning involving the cardiovascular, digestive, and hematological systems), with average accuracies of 73.2% and 71.5%, respectively. ERNIE Bot showed the most prominent variability in domain-specific performance: it achieved competitive accuracy in unit 1 (70.8%) comparable to that of DeepSeek but exhibited relatively lower performance in unit 4 (pediatrics and the female reproductive system; 68.3%) than the other models. Notably, ERNIE Bot recorded the highest single-unit accuracy (75.1% in unit 1; 2020) among all the Chinese models, although its overall performance was characterized by greater variability across different topic domains rather than year-to-year fluctuations.

Tongyi Qianwen demonstrated relatively stable performance, with scores consistently ranging between 401 and 451. The model showed particular strength in the early years (2018-2019) before experiencing a moderate decline in subsequent years. Notably, it maintained a narrow performance band of 413 to 420 during 2022 to 2024.

Doubao exhibited the most pronounced performance volatility among the 4 models. Starting at 401 in 2018, it reached its peak of 447 in 2019 before experiencing significant fluctuations (394 in 2021 and 417 in 2024). This variability suggests potential sensitivity to examination content changes or model architecture adjustments in certain years.

All models consistently exceeded the passing threshold (360), confirming their fundamental competence in medical knowledge assessment. Compared with the ChatGPT results in Figure 2, the Chinese LLMs showed comparable results to those of GPT-4 in the NMLE, with a score rate far exceeding that of GPT-3.5.

## Discussion

The findings of this study offer a nuanced understanding of how LLMs navigate the complexities of the Chinese NMLE, shedding light on their strengths, limitations, and implications for medical education.

### Model Performance Dynamics and Underlying Mechanisms

GPT-4’s consistent dominance over GPT-3.5 across all exam units—particularly its 14.39% accuracy advantage in unit 4 (female reproductive system and pediatrics)—reflects the tangible benefits of increased parameter scale and refined medical domain training. The model’s ability to maintain high scores in units requiring integrated clinical reasoning (eg, unit 3: cardiovascular and digestive systems) suggests that its pretraining on diverse medical literature and case studies enhanced contextual understanding. In contrast, GPT-3.5’s occasional failure to meet the passing threshold (eg, 329 points in 2019) underscores the critical role of model updates in medical accuracy as its performance improved significantly in later years with probable algorithmic refinements.

Among the Chinese LLMs, DeepSeek’s sustained excellence—scoring above 450 points in 4 consecutive years (2018‐2021)—can be attributed to its specialized architecture optimized for medical terminology and reasoning. DeepSeek’s 2024 score (465 points) following a 2-year decline aligns with the 2024 exam’s focus on basic medical sciences—a domain where the model consistently demonstrated strengths in our unit-specific analysis—rather than reflecting adaptive adjustments to changing exam standards. ERNIE Bot’s 2020 peak (473 points), surpassing even DeepSeek, highlights the potential of knowledge-enhanced models in specific assessment contexts, although its subsequent decline to 425 points in 2024 raises questions about knowledge base timeliness or training data diversity. Tongyi Qianwen’s steady but unremarkable performance (401‐451 points) suggests a balanced but generalized approach to medical knowledge, whereas Doubao’s volatility—spanning 394 to 447 points—may stem from its broader design focus on multidomain assistance rather than specialized medical precision.

A notable observation from this study is that the performance of all tested LLMs—including GPT-4 (highest average accuracy=80.67% in unit 4) and the top-performing Chinese model, DeepSeek (highest average accuracy=73.2% in unit 1)—did not exceed 80% across all NMLE units and years. This performance ceiling stems from 3 key factors consistent with broader medical AI research findings. First, the NMLE emphasizes high-order clinical reasoning (eg, integrating symptom clusters, laboratory results, and imaging findings for differential diagnosis) rather than just rote knowledge recall, and current LLMs struggle with such contextual nuance—evident in our study’s lower accuracy in unit 3 (focused on complex case analysis, eg, GPT-3.5’s 56.86% accuracy), where models often failed to prioritize key diagnostic indicators or account for comorbidities [15,16].

Second, training data timeliness and domain specificity limitations contributed to the sub-80% performance. Most LLMs have training data cutoffs that may exclude the latest clinical guidelines or emerging medical knowledge relevant to recent NMLE questions (eg, 2024 exam content). This was reflected in our study by GPT-4’s 24-point drop in unit 4 between 2023 and 2022 and DeepSeek’s fluctuating scores in unit 3 (cardiovascular and digestive systems), where questions on new pharmacotherapies or revised treatment algorithms likely fell outside the models’ training scope [17,18].

Third, the text-based input processing used in this study (to standardize comparisons) inevitably lost critical visual details from medical images (eg, chest x-ray infiltrate density and blood smear leukocyte patterns). Medical licensing exams increasingly rely on multimodal content to mirror real-world practice, and LLMs’ inability to directly interpret images reduces their engagement with such questions—observed in the lower accuracy across models in unit 2 (which includes pathological and radiological content, eg, GPT-4’s 69.05% accuracy vs 80.67% in unit 4) as text descriptions could not capture subtle visual cues for distinguishing conditions such as pneumonia and pulmonary edema [19].

### Implications for Chinese Medical Education

The consistent passing scores of all models (≥360 points) validate their utility as supplementary educational tools, particularly for foundational knowledge review. However, the performance gaps in specialized units reveal critical insights: GPT-4’s 80.67% accuracy in unit 4 contrasts with Doubao’s 59.87% average in the same unit, highlighting disparities in handling nuanced clinical scenarios. For Chinese medical curricula, DeepSeek and ERNIE Bot’s proficiency in Chinese medical ethics and regional clinical guidelines (eg, unit 1’s medical regulations) demonstrates their cultural and contextual relevance, making them more suitable for training students in China’s health care framework. This aligns with prior research emphasizing the importance of language-specific models in accurately interpreting culturally embedded medical practices.

### Limitations and Pathways for Advancement

This study’s focus on text-based questions—with all images and tables converted to text—represents a notable constraint as real-world medical practice frequently requires multimodal interpretation. Additionally, the exclusion of the 2023 to 2024 clinical guidelines (due to dataset limitations) may have impacted the models’ responses to emerging medical protocols. Future research should prioritize the following:

1. Multimodal model development—integrating computer vision algorithms to process radiology images or electrocardiograms, as seen in experimental medical AI systems
2. Dynamic knowledge integration—implementing real-time updates from sources such as the Chinese Medical Association’s guidelines to address timeliness gaps (discrepancies between the latest medical knowledge and guidelines and the information covered in model training data)
3. Human-LLM collaborative models—designing hybrid systems in which LLMs assist in diagnostic reasoning while human experts oversee complex decisions, reducing reliance on autonomous AI in high-stakes scenarios.

### Alignment of LLM Performance With Real-World and Literature Trends and Underlying Machine Learning Process Lacunae

The performance percentages of all tested LLMs align with trends reported in existing literature on medical AI and licensing exam assessments while also reflecting gaps consistent with documented limitations in machine learning (ML) processes for clinical tasks. Worldwide, studies on LLMs in medical licensing exams—such as evaluations on the US Medical Licensing Examination, Korean parasitology examinations, and prior NMLE analyses—show that even state-of-the-art models rarely exceed 85% accuracy, with most falling in the 60% to 80% range; this matches our findings, where GPT-4 (highest average accuracy=80.67%) and DeepSeek (73.2%) fit within this established spectrum. For instance, similar to our observation that GPT-3.5’s accuracy (68.5%) occasionally fell below the NMLE passing threshold, prior research on non–English-language medical exams has noted lower performance in models with less domain-specific fine-tuning, confirming consistency between our results and real-world model capabilities.

Notable lacunae in the ML process underlying these performance constraints include 3 key areas. The first is limited domain-specific fine-tuning for localized medical contexts. Most LLMs (including Chinese models) are pretrained on general medical corpora but lack targeted fine-tuning on region-specific clinical practices, guidelines, and NMLE-style question logic. For example, the NMLE emphasizes Chinese national medical regulations, regional disease prevalence (eg, specific infectious disease management protocols), and culturally aligned clinical decision-making—areas in which general ML training fails to deepen model understanding, leading to lower accuracy in unit 1 (which includes medical ethics and regulations) for models such as Doubao (average accuracy below 70%). The second key area is inadequate handling of low-resource clinical cases in training data. ML processes for LLMs rely heavily on large-scale, high-quality labeled data, but rare diseases, comorbid cases, and atypical symptom presentations (all critical to NMLE questions) are underrepresented in training datasets. This led to inconsistent performance in unit 3 (complex case analysis), where models such as GPT-3.5 (56.86% accuracy) frequently misdiagnosed rare comorbidities due to lack of training exposure. The third key area is static knowledge integration and absence of real-time learning. ML pipelines for current LLMs use fixed training data cutoffs (eg, pre-2023 data for many models), with no mechanism for real-time updates to incorporate new clinical guidelines, pharmacotherapies, or diagnostic criteria. This gap is reflected in our study’s 2023 to 2024 score fluctuations (eg, GPT-4’s 24-point drop in unit 4) as the models could not adapt to posttraining changes in medical knowledge—a limitation also highlighted in broader ML research on clinical AI.

### Broader Context and Ethical Considerations

The results also raise ethical questions about LLMs in medical assessment. While models such as GPT-4 and DeepSeek demonstrate impressive recall and reasoning, their lack of clinical experience may lead to oversights in rare cases or nuanced patient interactions. For instance, GPT-4’s occasional overconfidence in ambiguous questions (eg, 2023 unit 3 errors) highlights the risk of algorithmic bias. Therefore, educators must approach LLMs as diagnostic aids rather than substitutes for human judgment, emphasizing their role in enhancing efficiency rather than replacing professional expertise.

### Conclusions

This study provides the first comprehensive quantitative evaluation of LLMs in the NMLE, demonstrating that GPT-4 and Chinese models such as DeepSeek and ERNIE Bot can achieve reliable performance in structured medical assessments. GPT-4’s global generality and DeepSeek’s localized excellence each offer unique value: the former excels in cross-cultural medical reasoning, whereas the latter thrives in Chinese-specific medical contexts. However, all models face challenges in dynamic knowledge updating, multimodal processing, and contextual nuance—areas requiring urgent research attention. As AI continues to permeate health care education, these findings advocate for a hybrid approach: leveraging LLMs for scalable knowledge dissemination and formative assessment while preserving human-led training in clinical judgment and ethical decision-making. Ultimately, the integration of these technologies should enhance, not replace, the humanistic core of medical practice.

## Abbreviations

AI: artificial intelligence
LLM: large language model
ML: machine learning
NLP: natural language processing
NMLE: National Medical Licensing Examination
